# How to become leaders in psychiatry

**DOI:** 10.1192/j.eurpsy.2026.10261

**Published:** 2026-07-31

**Authors:** M. Asztalos

**Affiliations:** 1 Psychiatry, Aalborg University Hospital; 2 Department of Clinical Medicine, Aalborg University, Aalborg, Denmark

## Abstract

**Abstract:**

The topic of the ECP Programme, ‘Lessons in Leadership: How to become leaders in psychiatry,’ gives us a broad opportunity to explore different aspects of this important subject. Learning leadership skills is not prioritized in the curriculum, neither as medical students nor as a doctor during specialty training. In my presentation I will focus on Inner Leadership — the process by which a leader prioritizes reflection, motivation, transition and personal development in order to lead others more effectively.

**Disclosure of Interest:**

None Declared

